# Adrenal gland injury after blunt abdominal trauma: Two case series and review of the literature

**DOI:** 10.1016/j.ijscr.2020.01.021

**Published:** 2020-01-27

**Authors:** Konstantinos S. Papadopoulos, Konstantinos Strigklis, Kleoniki Kordeni, Panagiota Xaplanteri, Georgios Zacharis

**Affiliations:** a General Hospital of Patras, Surgery Department, Patras, Greece; b School of Health Rehabilitation Sciences, Department of Nursing, University of Patras, Patras, 26504, Greece

**Keywords:** Adrenal gland injury, Adrenal hematoma, Blunt abdominal trauma

## Abstract

**Introduction:**

Adrenal gland injury (AGI) is a rare form of trauma that is often misdiagnosed, especially if isolated.

**Presentation of case:**

Herein we describe two rare cases of adrenal hematoma (AH) and adrenal contusion due to blunt abdominal trauma. A 33-year-old Caucasian male who was transported to the emergency department after a low-speed motorbike accident and a 69-year old Caucasian female who fell from 50 cm height. Both where complaining of intense pain in the right flank. Neither were under anti-coagulant or anti-platelet therapy. In both patients thoracoabdominal CE-CT revealed the injury.

**Discussion:**

AGI is an uncommon finding in blunt trauma and isolated adrenal injuries usually result from low force accidents. Treatment is most commonly conservative, but depends on patient’s status. In Greece, to our knowledge, these are the first such cases reported in adults.

**Conclusion:**

Diagnosis needs high level of clinical suspicion.

## Highlights

• AGI is an uncommon finding in blunt trauma.
• Isolated adrenal injuries usually result from low force accidents.
• Treatment is most commonly conservative, but depends on patient’s status.
• Diagnosis needs high level of clinical suspicion.

## 1 Introduction

Adrenal gland injury (AGI) is a rarely described condition, able to happen in both high and low blunt thoracoabdominal trauma. It is usually presented in the form of isolated adrenal hematoma (AH), a very rare entity [1]. Herein we report two cases of isolated AGI due to blunt abdominal trauma. Those entities are described rarely in literature. In Greece, to our knowledge, they are the first such cases reported in adults. The work has been reported in line with the SCARE criteria [2].

### 1.1 Case 1

A 33-year-old Caucasian male was transported to the emergency department after a low-speed motorbike accident. He had a free medical record with occasional tobacco use. On presentation, the patient had normal vital signs: Blood pressure: 133/91 mmHg, heart rate: 72bpm, peripheral oxygen saturation (SpO2): 100 %, Temperature 36,8 °C. He complained of deep right upper quadrant and right flank at rest and right upper limb pain, without evidence of ecchymosis or lacerations on his chest or abdomen. Some superficial abrasions were visible on his right elbow and his right lower quadrant. Physical examination revealed mild tenderness to deep palpation in his right upper quadrant and right flank.

**Fig. 1:**
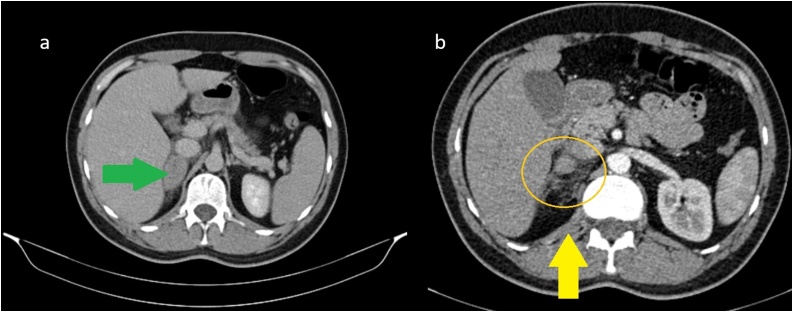
Oval, well-defined lesion (4.2 × 2.7) with high density (green arrow) and crus thickening (yellow arrow), indicative of acute AH in the initial abdominal CT. No contrast extravasation is noticed. Periadrenal fat stranding appears inferiorly (circle).

**Fig. 2:**
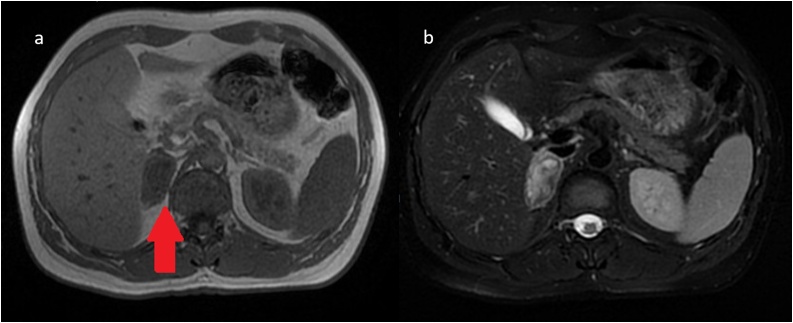
7-day follow-up MRI shows hypointense enlargement of the right adrenal (arrow) in T1 and an anomeogenous gland with hyperintense elements possibly haemorrhagic in diffuse weighted imaging (DWI).

**Fig. 3:**
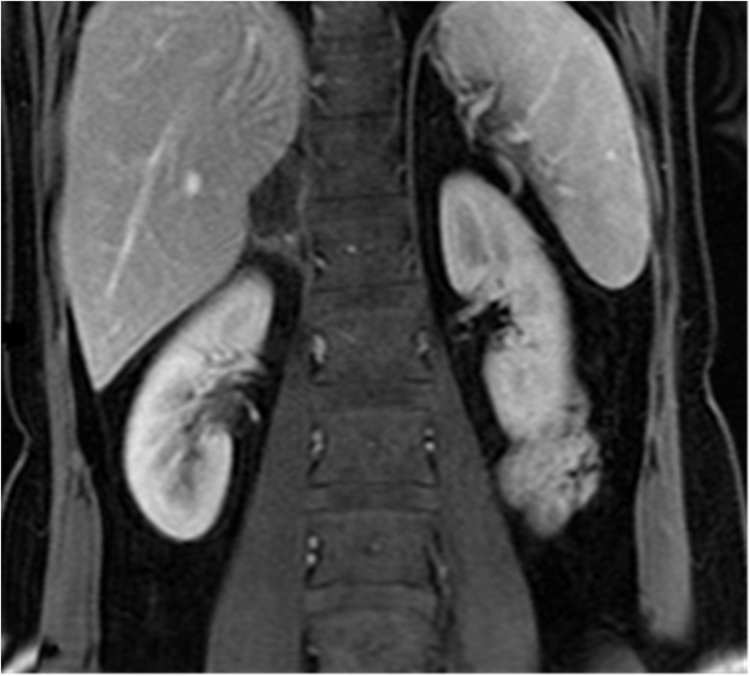
Close affiliation of the structures is shown through the first follow-up MRI, posterior to the injured adrenal gland seems not only compressed between the liver and the vertebrae, but also reshaped in contact with the latter.

**Fig. 4:**
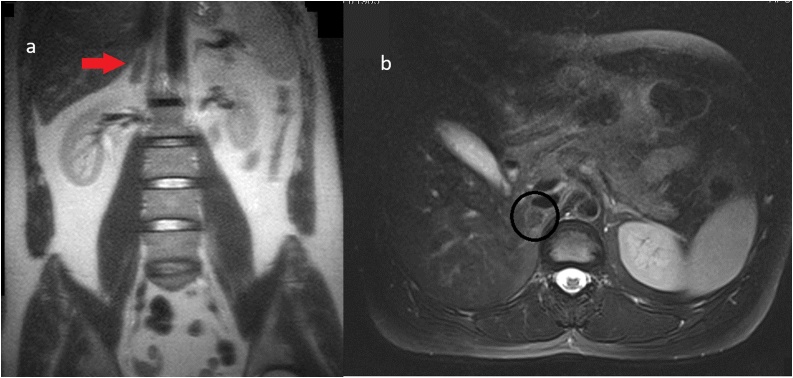
60-day follow-up reveals a greatly reduced lesion typical of a chronic AH (arrow) with hypointense T1 signal and iso- to hypointense lesion with slight hyperintense capsule in T2 sequence (circle) with maximum diameter at 1.3 cm.

There were no abnormal signs on his blood analysis, metabolic or coagulation panel. Urinalysis revealed mild microscopic hematuria (20–26 erythrocytes per field vision). Chest, spine and pelvis plain radiography was negative for fractures or any other abnormal findings. A focused assessment sonography for trauma (FAST) performed by a clinical radiologist in the Emergency Department revealed no evidence of trauma. An abdominal contrast-enhanced computed tomography scan (CE-CT) was performed due to persistence of pain and high clinical suspicion. The CT revealed round enlargement of the right adrenal (4.2 × 2.7 cm) with increased density (60-70HU), periadrenal fat stranding and right diaphragmatic crus thickening without extravasation of the intravascular contrast. No injuries from other organs were spotted and the diagnosis of an isolated right adrenal hematoma (AH) was made (Fig. 1). As a result, he was admitted to the clinic for preservative treatment and further monitoring. He had stable vitals and body temperature throughout his hospitalization period and gradually improving pain after intravenous analgesia. He was discharged with oral antibiotics and analgesia for 6 days. A follow-up MRI scheduled in 7 days, showed no size reduction and acute AH characters with signs of possible active hemorrhage (Fig. 2), whereas, close affiliation of the structures posterior to the injured adrenal gland shows compression and chance of shape of the adrenal between the liver and the vertebrae (Fig. 3). The patient was free of symptoms and his urinalysis, metabolic panel and adrenocorticotropic hormone (ACTH) stimulation test performed were normal. New follow-up with abdominal MRI scheduled after 60 days revealed partial resolution of the AH, while the lesion that remained had elements of a chronic hematoma (Fig. 4).

### 1.2 Case 2

**Fig. 5:**
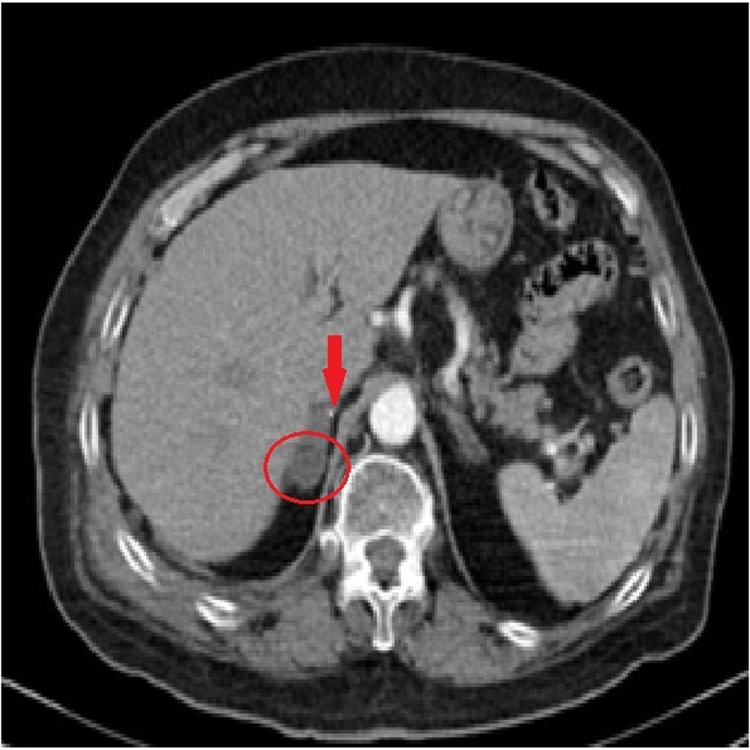
CE-CT reveals a contrast-enhanced dot-shaped focal area anterior to the gland (arrow).

**Fig. 6:**
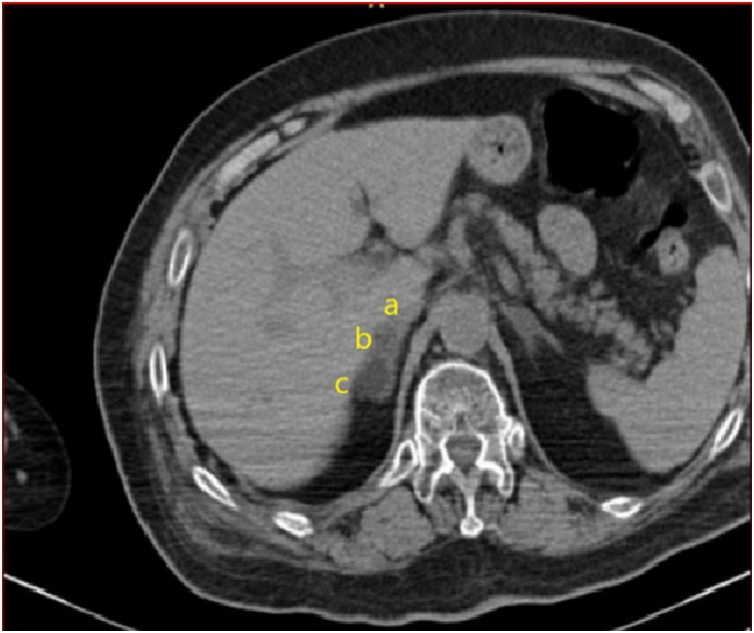
Plain abdominal CT follow-up imaging after three days shows a distinct hypodense area of the non-traumatic oval lesion and a smaller posterior hyperdense area (55 HU) of the gland close to the liver. Notice the three different density areas around the gland (a,b,c).

A 69-year old Caucasian female was admitted to the emergency department due to fall from 50 cm height and she was complaining of intense pain in her right flank around the impact area. The examination revealed pain in the lower right thoracic area and right upper quadrant in superficial and deep palpation with no sign of abrasions or ecchymosis. Her vital signs, on presentation, were slightly abnormal (Blood pressure: 150/83 mmHg, Heart rate: 84 bpm, SpO2: 97 %, Body Temperature: 36.7 °C). The patient’s medical history consisted of hypertension under medication and chronic obstructive pulmonary disease (COPD) due to smoking. She did not mention anti-coagulant or anti-platelet use. Chest radiograph showed minor right pleural effusion without rib fractures and FAST did not reveal any evidence of trauma. There were no abnormal findings from her blood analysis, metabolic/coagulation panel, and urinalysis. The patient constantly complained about pain in her right upper quadrant and right flank even after the administration of analgesics. A thoracoabdominal CE-CT was performed to evaluate the origin of pleural effusion and abdominal pain. The CT confirmed the pleural fluid diagnosis as well as mentioned a small round lesion of variable attenuation with a very small focal hyperdense area on the right adrenal, with no active extravasation (Fig. 5). No other traumatic injuries from the chest or abdomen were described. The patient was admitted to the clinic for monitoring. She remained hemodynamically stable but complained of pain and difficulty in deep breath even with IV analgesia. Therefore, a thoracoabdominal CT scan was scheduled. The right adrenal lesion appeared as a more well-defined hypodense oval mass, possibly adenoma, while the rest of the gland was slightly enlarged and hyperdense (Fig. 6). The possibility of mild adrenal contusion was discussed. The pleural effusion remained unchanged and treated conservatively. No other evidence of trauma was concluded from the rest of the radiographic examination. Hyponatremia and hypokalemia remained even after fluid and electrolyte administration but otherwise she had normal lab values. The patient’s pain improved after three days and was discharged after a 5-day hospitalization with oral antibiotic and pain medication. Unfortunately, the patient was lost on scheduled follow-up.

## 2 Discussion

Adrenals are small retroperitoneal glands directly below the diaphragm attached to kidneys. They are attached to the crura of the diaphragm by the renal fascia. The right adrenal, is more posteriorly than the left, has the shape of a pyramid and is related anteriorly to the right lobe of the liver and the inferior vena cava (IVC) [3].

The left adrenal is crescent in shape and surrounded by the stomach and the spleen. It is located further from the spine than the right. The right adrenal vein medially with the spinal column, whereas the left adrenal vein joins the left renal vein. As a result the right adrenal vein is more susceptible to contusions and edema [3]. In most cases described in literature the right adrenal is more likely to undergo AGI [4,5]. In both our patents the trauma was inflicted on the right adrenal.

Force application in a lateral manner provoking minimal damage seems to be the cause of AGI [1]. The first patient underwent a low-speed motorbike accident, whereas the second fall from 50 cm height. Similar reports for children patients have revealed that some part of the adrenal gland was intact [1,6].

Adrenal trauma diagnosis is made solely by imaging tools because of the lack of indicative examinations. Clinical appearance includes vague back pain and physical or laboratory examination might reveal hyper- or hypo-tension, leukocytosis, microscopic hematuria and mild electrolyte disorders [7]. FAST is the most frequently used tool by surgeons and radiologists for trauma patients and can identify AGI while examining the Morison’s pouch or the splenorenal recess. However most of the cases are diagnosed with abdominal CT by radiologists as many lesions could not be recognized and evaluated by FAST or the patient suffered from multiple organ injuries that required further imaging [8].

Ultrasound sonography (U/S) can reveal clinically significant AGI, but can also fail due to small size of the glands and the experience of the examiner. The method of choice for the diagnosis of adrenal trauma is Computed Tomography with intravascular contrast (CE-CT) [9]. In both our patients, diagnosis was based on high clinical suspicion due to the persistence of pain, and was confirmed by CT scan.

MRI use is uncommon in emergency medicine but serves best for follow-up purposes, as we did in our first case.

The management of patients with AGI depends on the patient status, the severity of trauma and the associated injuries. Stable patients with AGI are treated conservatively with monitoring in most of the cases whereas angiographic embolization can be performed in hemodynamically unstable cases with active extravasation or patients that open surgery is contraindicated. Right inferior artery is embolized in most of the cases. Complications from embolization like gland necrosis, adrenal insufficiency or recurrent bleeding are almost abscent [10]. In cases of uncontrolled bleeding or lack of angiography, suture hemostasis or adrenalectomy can be performed via laparotomy with non-significant changes in patient mortality.^10^

Close follow-up after conservative treatment is common, as in most cases total or partial resolvement of AH happens after 2–4 weeks [1,4,5]. Our first patient was punctual on follow-up, whereas the second one missed her scheduled follow-up. In addition, the main question mark in borderline situations as in Case 2, that was to clarify whether or not the AH occurred on a pre-existing adrenal lesion or misdiagnosed as adrenal adenoma with similar cases reported in the past [11].

In adult patients in Greece, to our knowledge, there are no former described cases of adrenal blunt injury. Some cases in children have been described by Roupakias et al. [12].

## 3 Conclusion

AGI is an uncommon finding in blunt trauma and isolated adrenal injuries usually result from low force accidents. Treatment is most commonly conservative, but depends on patient’s status. Diagnosis needs high level of clinical suspicion.

## Sources of funding

This research was supported by funding of the Department of General Surgery St. Andrew’s General Hospital, Patras, Greece.

## Ethical approval

The study has been approved by the Ethics committee of St. Andrew’s General Hospital, Patras, Greece, approval number 41541.

## Consent

Written informed consent was obtained from the patient for publication of this case report and accompanying images. A copy of the written consent is available for review by the Editor-in-Chief of this journal on request”.

## Author contribution

All authors have contributed in study concept and design, data collection, data analysis and interpretation.

## Registration of research studies

None.

## Guarantor

All authors

## Provenance and peer review

Not commissioned, externally peer-reviewed

## Declaration of Competing Interest

The authors declare that they have no competing interests.
